# PD-L1 expression across molecular driver subsets in non-small cell lung carcinoma: a 299-case real-world thoracic oncology cohort

**DOI:** 10.1186/s12885-026-16599-7

**Published:** 2026-07-30

**Authors:** Arzu Taşdemir, Hatice Karaman, Nihal Kiremit, Murat Levent Güneş, Ender Doğan, İbrahim Ethem Özsoy

**Affiliations:** 1 Department of Pathology, Kayseri City Hospital, Şeker Mah. Muhsin Yazıcıoğlu Bulvarı No. 77, Kocasinan, Kayseri 38080 Turkey; 2 Department of Medical Oncology, Kayseri City Hospital, Kayseri, Turkey; 3 Department of Thoracic Surgery, Kayseri City Hospital, Kayseri, Turkey

**Keywords:** Biomarkers, tumor, Carcinoma, non-small-cell lung, KRAS, Molecular diagnostic techniques, Programmed cell death 1 ligand 1

## Abstract

**Introduction:**

PD-L1 immunohistochemistry and molecular driver testing are complementary components of non-small cell lung carcinoma (NSCLC) evaluation. We examined the distribution of PD-L1 tumor proportion score (TPS) across routinely detected molecular driver subsets in a consecutive real-world thoracic oncology cohort.

**Methods:**

We retrospectively reviewed 299 consecutive NSCLC cases that underwent clinical targeted next-generation sequencing (NGS) between 2023 and 2025. Archived VENTANA SP263-stained PD-L1 slides were re-reviewed by two pathologists and categorized as TPS < 1%, 1–49%, or > = 50%. Driver-association analyses were restricted to PD-L1-tested tumors with an evaluable, mutually exclusive primary driver category; cases with indeterminate DNA-driver status were excluded. Prespecified KRAS and EGFR contrasts used reproducible 2 × 2 methods, with univariable Firth regression as a sparse-cell sensitivity analysis.

**Results:**

PD-L1 results were available for 237 tumors. TPS was < 1% in 63 cases (26.6%), 1–49% in 108 cases (45.6%), and > = 50% in 66 cases (27.8%). Among 223 PD-L1-tested tumors with an evaluable, mutually exclusive primary driver category, KRAS-mutant tumors were more frequently PD-L1 high than the other evaluable categories (24/54 [44.4%] vs. 35/169 [20.7%]; OR, 3.06; 95% CI, 1.59–5.89; *p* = 0.001). EGFR-mutant tumors showed a lower PD-L1-high frequency, but this observation was based on only two PD-L1-high events (2/26 [7.7%] vs. 57/197 [28.9%]; OR, 0.20; 95% CI, 0.05–0.89; *p* = 0.019). Eleven cases with indeterminate DNA-driver status were excluded from driver-frequency and association analyses.

**Conclusion:**

PD-L1 high expression was enriched among KRAS-mutant NSCLC in this diagnostic cohort, whereas EGFR-mutant tumors showed a directionally lower but sparse-data-limited PD-L1-high pattern. These findings support integrated interpretation of PD-L1 and molecular driver results in routine practice, but they should be viewed as biomarker-distribution evidence rather than proof of immunotherapy benefit or prognosis.

**Supplementary Information:**

The online version contains supplementary material available at https://doi.org/10.1186/s12885-026-16599-7.

## Introduction

The diagnostic evaluation of non-small cell lung carcinoma (NSCLC) has evolved from morphology-centered classification to an integrated thoracic oncology workflow that combines histologic subtype, immunohistochemistry, programmed death-ligand 1 (PD-L1) tumor proportion score (TPS), and molecular driver testing [1–4]. This integrated approach is particularly important in small biopsies, where tissue stewardship and timely sequencing of immunohistochemical and molecular assays directly influence the completeness of the diagnostic report.

PD-L1 immunohistochemistry is routinely interpreted using clinically relevant TPS categories, commonly < 1%, 1–49%, and > = 50%. Although these thresholds are familiar in practice, PD-L1 assessment can be affected by pre-analytic variables, assay platform, antibody clone, staining protocol, tumor heterogeneity, and reader interpretation. Standardized scoring and careful correlation with molecular findings are therefore central to reproducible diagnostic pathology and thoracic oncology decision-making [5–7].

Molecularly defined NSCLC subsets differ in immune phenotype. Meta-analyses and large real-world cohorts from different populations have reported higher PD-L1 expression in KRAS-mutant tumors and lower PD-L1 expression in EGFR-mutant tumors [8–12]. These observations are broadly consistent across studies, but the magnitude of association can vary with population structure, smoking prevalence, assay platform, testing breadth, specimen pathway, and the handling of missing PD-L1 results.

KRAS-mutant disease is biologically heterogeneous, and co-alterations such as STK11/LKB1, KEAP1/NFE2L2, and TP53 can reshape the immune microenvironment and influence response to PD-(L)1 blockade [13–17]. EGFR-mutant and ALK-rearranged tumors also require cautious interpretation of PD-L1 results because high PD-L1 expression alone does not establish expected benefit from immune checkpoint inhibition in driver-positive disease [18, 19]. ALK is mentioned here for conceptual completeness; only three ALK-fusion cases were identified in the present cohort, so no ALK-specific PD-L1 analysis was attempted.

These issues are directly relevant to thoracic oncology because PD-L1 and NGS results may enter clinical discussion before all biomarker components are available [20, 21]. In routine practice, PD-L1 may be reported before complete DNA/RNA results are finalized. A cohort that describes how TPS categories distribute across driver subsets can therefore help clinicians and laboratories avoid overinterpretation of an isolated biomarker result.

Our aim was therefore practical rather than discovery-driven. In a consecutive, regionally grounded Turkish diagnostic cohort, we examined how SP263 TPS rescoring, specimen pathway, PD-L1 test availability, targeted DNA/RNA workflow, and KRAS variant heterogeneity intersect during routine reporting. This allows the results to be read alongside prior meta-analyses, large Asian cohorts, and international real-world series while drawing attention to diagnostic-pathway details that are not always visible in those datasets [8–12, 22].

## Methods

### Study design and case selection

This retrospective archive-based study included consecutive patients with NSCLC who underwent clinical molecular testing at Kayseri City Hospital between 2023 and 2025. Cases were identified from the institutional pathology information system and molecular testing registry. The analytic export contained 306 specimen-level records. Repeated specimens were linked at the patient level using the cohort identifiers; the earliest eligible tumor was retained for clinicopathologic, primary driver, and PD-L1 analyses, while DNA and RNA testing flags were consolidated across all linked specimens solely to describe whether each patient had undergone each assay modality. This process yielded 299 unique patients.

Seven patients had no mutually exclusive primary category because of concomitant drivers (EGFR + KRAS, *n* = 3; KRAS + BRAF, *n* = 2; ERBB2 + BRAF, *n* = 1) or isolated NRAS mutation (*n* = 1). A second-stage assay-provenance audit identified 11 additional patients whose DNA-driver status could not be established: 10 had a negative RNA fusion result without a documented DNA panel, and one carried a KRAS G12F label but had only an RNA-fusion testing flag and no retained report or DNA-panel flag establishing a KRAS-capable assay. Because KRAS was not a target of the documented RNA panel, that label was not retained as an analytically verified KRAS event. These 11 patients remained in the overall cohort and PD-L1 distribution but were classified as DNA-driver-status indeterminate and excluded from driver-frequency and association analyses. Thus, the mutually exclusive molecularly evaluable driver set contained 281 patients. The primary PD-L1 association set contained 223 tumors after excluding nine PD-L1-tested indeterminate cases and five PD-L1-tested concomitant/NRAS cases. The case-selection and analysis sets are summarized in the Supporting Information (see Additional file 1) (Text S1 and Table S2).

Histologic classification was based on routine diagnostic workup according to contemporary WHO principles. Specimen type was recorded as biopsy, resection/excision specimen, or externally submitted paraffin block. Smoking history was categorized as never, former, or current when documented; otherwise, it was coded as missing/unknown. Stage, systemic treatment sequence, immune checkpoint inhibitor exposure, and survival outcomes were not uniformly available. Accordingly, all analyses were interpreted as biomarker-distribution analyses.

### Molecular testing

Tumor-rich formalin-fixed paraffin-embedded (FFPE) tissue areas with an estimated tumor cellularity of at least 20% were selected for nucleic acid extraction, with tumor content estimated by a pathologist on hematoxylin and eosin-stained sections. Molecular testing was performed as part of routine clinical care.

Two DNA panel versions were used during the study period. The 9-gene somatic lung cancer DNA panel interrogated ALK, MET, ROS1, RET, ERBB2, BRAF, EGFR, KRAS, and NRAS. The comprehensive 30-gene somatic lung cancer DNA panel interrogated FGFR2, MET, CDK6, SOD2, ESR1, ROS1, RICTOR, TERT, KIT, PDGFRA, PIK3CA, MYD88, ERBB4, IDH1, IDH2, ALK, STK11, ERBB2, TP53, MAP2K1, AKT1, CDK4, GAPDH, PTEN, DDR2, NTRK1, BRAF, EGFR, KRAS, and NRAS. EGFR and KRAS were represented in both DNA panel versions. STK11 and TP53 were included only in the 30-gene panel; KEAP1 was included in neither documented DNA panel, and RET was included in the 9-gene DNA panel and RNA workflow but not in the documented 30-gene panel. After patient-level consolidation, a documented DNA panel was present in 284/299 patients (95.0%): 52 (17.4%) had the 9-gene panel and 232 (77.6%) had the 30-gene panel; the two DNA-panel categories were mutually exclusive. All 65 analytically verified primary KRAS-mutant tumors had a documented KRAS-capable DNA panel (16 with the 9-gene panel and 49 with the 30-gene panel). Exact assay targets and counts are provided in Supporting Table S1.

For biopsy specimens, testing generally followed a clinician-directed sequential strategy: DNA-based NGS first, with targeted RNA fusion sequencing added selectively when requested by the treating team or pathologist and tissue was sufficient, most often when a fusion-driven tumor remained a clinical consideration after DNA testing [23, 24]. RNA testing was not performed reflexively for all adenocarcinomas. The 25-gene RNA fusion panel interrogated NOTCH2, PDGFRA, TERT, PDGFRB, MYB, ETV1, EGFR, MET, BRAF, NRG1, RET, ETV6, NUTM1, ETV4, BRD4, ERG, EWSR1, TFE3, ALK, FGFR2, FGFR3, NTRK1, NTRK2, NTRK3, and ROS1; it did not interrogate KRAS. After patient-level consolidation, RNA fusion testing was documented in 37/299 patients (12.4%): 22 (7.4%) had both DNA and RNA testing, and 15 (5.0%) had RNA testing without a documented DNA panel. Of these 15 RNA-only patients, four had an NTRK2 fusion detected and were retained as NTRK2-positive; 10 had a negative RNA fusion result and were classified as DNA-driver-status indeterminate; the remaining patient was the unsupported KRAS G12F label described above and was also classified as indeterminate. A negative RNA fusion result without DNA testing was never classified as driver-negative (Supporting Table S1).

DNA was extracted from FFPE tissue using the QIAamp DNA FFPE Advanced Kit (QIAGEN, Hilden, Germany) according to the manufacturer’s protocol. Targeted library preparation and enrichment used institutionally validated workflows. Libraries were sequenced on Illumina MiSeq (Illumina, San Diego, CA, USA) and/or Element Biosciences platforms (Element Biosciences, San Diego, CA, USA). Both sequencing platforms were used within validated clinical workflows, the same 5% reportable minimum variant allele frequency threshold was applied to issued reports, and no systematic platform-related discrepancy was recorded during routine report review. The retrospective analytic export did not retain complete platform-specific case counts or run-level data sufficient for a formal platform-level variant-call comparison. Run-level quality metrics and minimum coverage requirements were reviewed before a sample was considered evaluable.

Routine clinical analysis was performed using CLC Genomics Workbench (v22.0.2; QIAGEN) and Clinical Insight Interpret (v9.3.0.20240531; QIAGEN). Variants were interpreted using a tier-based somatic framework consistent with AMP/ASCO/CAP principles. Driver-negative status required at least one documented DNA panel and no reportable alteration within the targets and alteration classes actually assayed; it does not indicate genome-wide molecular negativity. RNA-only fusion-negative patients were classified as DNA-driver-status indeterminate rather than driver-negative. Because panel breadth differed, fusion status and co-mutation context were interpreted only within the assay actually performed.

### PD-L1 immunohistochemistry and scoring

PD-L1 testing availability and available original clinical PD-L1 report information were abstracted from institutional pathology records. PD-L1 results were available for 237 of 299 cases. Reasons for missing PD-L1 results were not systematically recorded, but likely reflected tissue limitations, externally submitted block logistics, and/or absence of a clinical testing request. Cases without available PD-L1 results were excluded from PD-L1 association analyses, and potential selection effects were explored by comparing tested and untested cases.

PD-L1 immunohistochemistry had been performed in routine practice on the VENTANA BenchMark ULTRA platform using the VENTANA PD-L1 (SP263) assay (Roche Diagnostics, Basel, Switzerland). In the routine diagnostic pathway, PD-L1 immunohistochemistry and molecular testing were generally ordered during the same diagnostic episode, but their exact sequence could differ by specimen type, tissue adequacy, clinical request, and whether the material was an in-house specimen or an externally submitted block. Exact test-order dates were not available for all cases.

For the present study, archived stained slides from the 2023–2025 study period were retrieved from the institutional slide archive and re-evaluated. The exact interval between staining and re-review was not recorded for every slide, and storage conditions were those of the routine diagnostic slide archive. TPS was defined as the percentage of viable tumor cells with partial or complete membranous staining of any intensity and was categorized as < 1%, 1–49%, or > = 50%. Two pathologists assigned TPS categories using prespecified SP263 interpretation criteria. Whole-slide assessment, rather than hotspot-only estimation, was used; staining adequacy was checked with external and internal controls. Discrepancies affecting TPS category were resolved by consensus.

Available original clinical reports were checked to document routine PD-L1 reporting and, when possible, to determine whether original category-level results had been retained. However, the analytic dataset did not contain paired original clinical TPS categories and consensus rescored TPS categories in separate fields for all cases, and independent pre-consensus category-level rescoring data were not retained. Formal clinical-versus-rescored concordance statistics and interobserver kappa could therefore not be calculated. The consensus rescored TPS category was used for all primary analyses. Accordingly, slide re-review should be interpreted as standardized two-pathologist consensus categorization rather than as quantitative evidence of improved interobserver reproducibility.

**Fig. 1:**
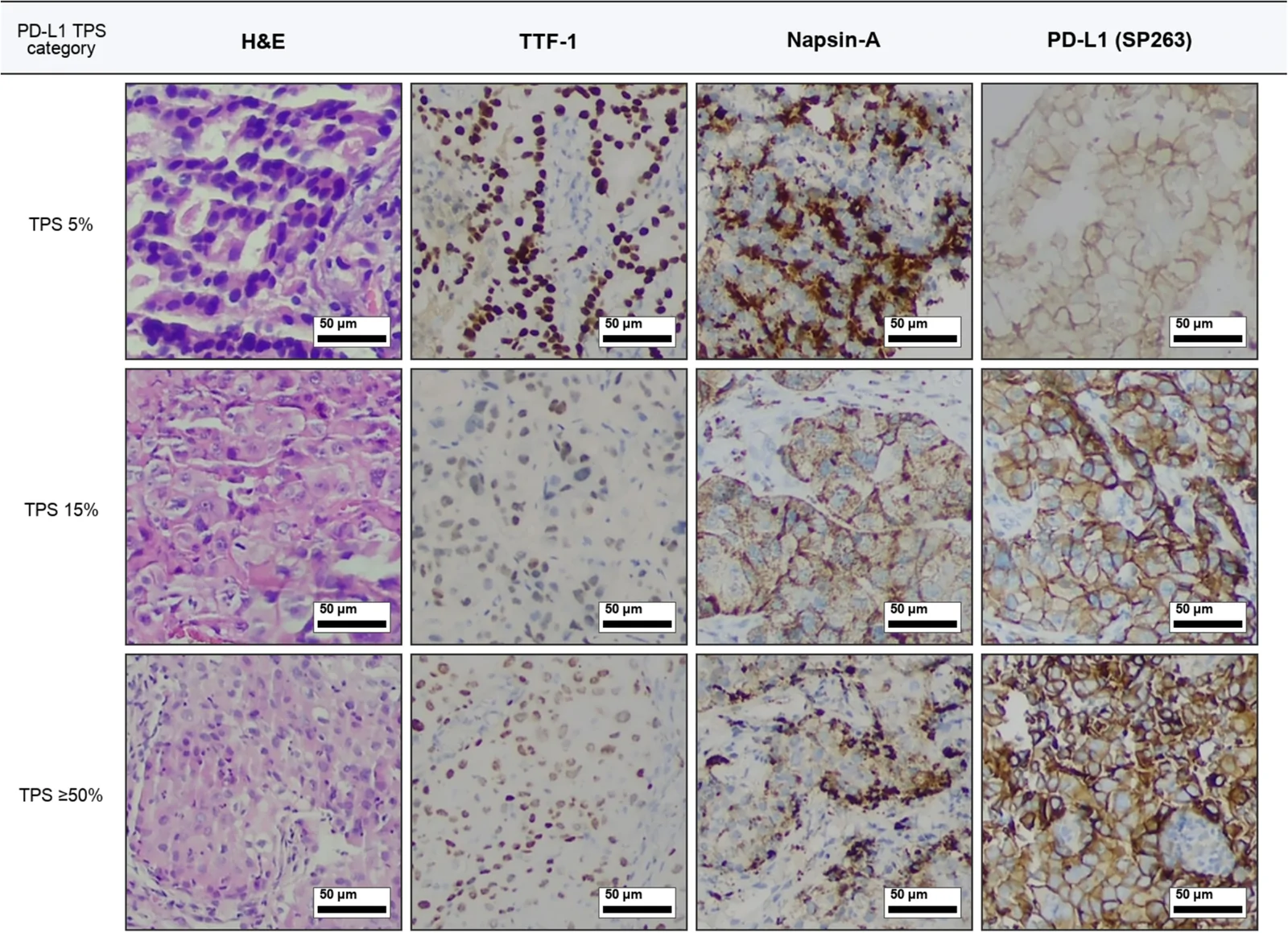
Representative histopathology and immunohistochemistry across PD-L1 tumor proportion score (TPS) categories. Columns show H&E tumor morphology, TTF-1, Napsin-A, and PD-L1 (SP263). Rows show representative cases with TPS approximately 5%, 15%, and > = 50%. Brown staining indicates positive immunoreactivity. Scale bars: 50 μm

For the representative microscopic panel (Fig. 1), archived de-identified H&E and immunohistochemistry images were assembled as a composite figure. Borders demarcate separate image fields. Scale bars are indicated in the figure.

### Statistical analysis

All analyses used available cases. Baseline characteristics of PD-L1-tested and untested cases were compared using Welch’s t-test for age and chi-square or Fisher’s exact tests for categorical variables. Potential selection related to PD-L1 availability was evaluated descriptively. Aggregate specimen-type category counts were retained, but a patient-level specimen-type source linked to the corrected analytic export was not available; therefore, no multivariable availability model was retained, and the previous model could not be independently reconstructed.

For 2 × 2 comparisons of PD-L1 high expression (TPS > = 50%), Fisher’s exact test was used when any cell count was < 5; otherwise, Pearson’s chi-square test with Yates continuity correction was applied. Odds ratios (ORs) with conventional log-Wald 95% confidence intervals (CIs) were calculated from 2 × 2 contingency tables. Because PD-L1 high expression was not rare, ORs were interpreted as association measures rather than relative risks; unadjusted prevalence ratios were provided descriptively. The prespecified contrasts compared the mutually exclusive primary KRAS category with all other molecularly evaluable mutually exclusive categories and the primary EGFR category with all other evaluable categories. Nine PD-L1-tested indeterminate cases and five PD-L1-tested concomitant/NRAS cases were excluded, leaving 223 tumors for these contrasts (Supporting Table S2).

As a sparse-cell sensitivity analysis, univariable Firth penalized logistic regression was performed for the prespecified binary KRAS and EGFR contrasts using the same 2 × 2 data; profile-likelihood CIs and penalized likelihood-ratio p values are reported in Supporting Table S5. No adjusted PD-L1-high model was retained after the assay-provenance audit because the corrected case-level molecular set could not be joined to a retained patient-level specimen-type source. Carrying forward the previous adjusted estimates would therefore have mixed corrected driver classifications with a model that could not be reproduced. The final analysis was intentionally limited to fully reconstructable unadjusted and Firth estimates (Supporting Tables S2 and S5).

The prespecified KRAS and EGFR contrasts were distinguished from KRAS variant-level summaries, adenocarcinoma-restricted analyses, smoking-subset descriptions, and other subgroup analyses, which were exploratory. No adjustment for multiple comparisons was applied; nominal p values were interpreted alongside effect sizes, CIs, and event counts.

Smoking was not included in modeling because the retained patient-level export documented smoking history for only 69/299 patients (23.1%); smoking-related results were therefore descriptive, and residual confounding remains possible.

### Ethics

This retrospective study was performed in accordance with the Declaration of Helsinki and was approved by the Kayseri City Hospital Non-Interventional Clinical Research Ethics Committee (Decision No. 767; 03 February 2026). The requirement for informed consent was waived because of the retrospective design and use of de-identified archived diagnostic material.

## Results

### Clinicopathologic characteristics

The mean age of the cohort was 65.6 +/- 9.1 years (range, 29–88 years). Men accounted for 81.6% of cases (*n* = 244) and women for 18.4% (*n* = 55). Histologic subtypes were adenocarcinoma in 61.2%, squamous cell carcinoma in 21.1%, NSCLC-NOS in 15.1%, and other or rare histologies in 2.7% (Table 1). Specimen types included biopsy (*n* = 169, 56.5%), resection/excision (*n* = 69, 23.1%), and externally submitted paraffin block (*n* = 61, 20.4%).

**Table 1:** Clinicopathologic characteristics of the overall cohort

Characteristic	*n* (%) or value
Age, years, mean +/- SD (range)	65.6 +/- 9.1 (29–88)
Male sex	244 (81.6)
Female sex	55 (18.4)
Adenocarcinoma	183 (61.2)
Squamous cell carcinoma	63 (21.1)
NSCLC-NOS	45 (15.1)
Other/rare histology	8 (2.7)
Biopsy specimen	169 (56.5)
Resection/excision specimen	69 (23.1)
Externally submitted paraffin block	61 (20.4)
Current smoker	45 (15.1)
Former smoker	17 (5.7)
Never smoker	7 (2.3)
Smoking history missing/unknown	230 (76.9)

Values are n (%) unless otherwise stated. Age is given as mean +/- SD

A second patient-level testing-flag audit identified 306 specimen-level entries and 299 unique patients after de-duplication. After consolidating assay flags across repeated specimens, 52 patients (17.4%) had the 9-gene DNA panel, 232 (77.6%) had the 30-gene DNA panel, and 37 (12.4%) had the 25-gene RNA fusion panel. Overall, 284 patients (95.0%) had a documented DNA panel, 22 (7.4%) had both DNA and RNA testing, and 15 (5.0%) had RNA testing without a documented DNA panel. The RNA-only group comprised four NTRK2-positive patients and 11 patients with indeterminate DNA-driver status.

### Driver alterations and KRAS variant spectrum

After excluding seven concomitant/NRAS cases and 11 patients with indeterminate DNA-driver status, 281 patients had a molecularly evaluable mutually exclusive primary driver category. KRAS was the most frequent alteration (23.1%), followed by EGFR (13.5%). Less common alterations included BRAF (2.1%), ERBB2/HER2 (1.8%), NTRK2 fusion (1.4%), ALK fusion (1.1%), MET (0.4%), RET fusion (0.4%), and FGFR3-TACC3 fusion (0.4%). Driver-negative status, which required documented DNA testing, accounted for 55.9% (Table 2). All NTRK rearrangements detected in this cohort involved NTRK2.

**Table 2:** Primary driver alterations among molecularly evaluable cases with a mutually exclusive primary driver category (*n* = 281)

Primary driver alteration	*n*	%
KRAS	65	23.1
EGFR	38	13.5
BRAF	6	2.1
ERBB2/HER2	5	1.8
NTRK2 fusion	4	1.4
ALK fusion	3	1.1
MET	1	0.4
RET fusion	1	0.4
FGFR3-TACC3 fusion	1	0.4
Driver-negative	157	55.9

Eleven cases with indeterminate DNA-driver status and seven concomitant/NRAS cases were excluded. Driver-negative required documented DNA testing and no reportable alteration within the tested targets/alteration classes; it does not indicate genome-wide molecular negativity

Within the 65 analytically verified primary KRAS-mutant tumors, the most common variants were G12C (40.0%) and G12V (24.6%), followed by Q61H (9.2%) and G12D (7.7%) (Table 3). Sixteen had the 9-gene DNA panel and 49 had the 30-gene DNA panel. PD-L1 high expression was observed in 10/22 PD-L1-tested G12C tumors and 8/16 PD-L1-tested G12V tumors. Variant-level analyses were descriptive and no allele-specific inferential claim was made.

**Table 3:** KRAS variant spectrum among analytically verified primary KRAS-mutant tumors (*n* = 65)

KRAS variant	*n*	%
G12C	26	40.0
G12V	16	24.6
Q61H	6	9.2
G12D	5	7.7
G13D	2	3.1
G12A	2	3.1
G12F	1	1.5
Other	7	10.8

KRAS variant data are descriptive only. Percentages are calculated among KRAS-mutant tumors

### PD-L1 testing availability and TPS distribution

PD-L1 testing was available for 237 of 299 tumors (79.3%). Testing frequency differed by specimen type and was lower for externally submitted paraffin blocks (38/61, 62.3%) than for biopsies (140/169, 82.8%) or resection/excision specimens (59/69, 85.5%; *p* = 0.001). This pattern suggests that PD-L1 availability was influenced by specimen logistics rather than biology alone. The detailed tested-versus-untested comparison is provided as Supplementary Table S4.

PD-L1 availability was 54/65 (83.1%) in the KRAS category, 26/38 (68.4%) in the EGFR category, and 143/178 (80.3%) in the other molecularly evaluable mutually exclusive categories (overall *p* = 0.180). Nine of 11 indeterminate cases and five of seven concomitant/NRAS cases had PD-L1 results but were not included in this category comparison (Supporting Table S4).

Within the PD-L1-tested subset, TPS was < 1% in 63 tumors (26.6%), 1–49% in 108 tumors (45.6%), and > = 50% in 66 tumors (27.8%) (Table 4).

**Table 4:** PD-L1 tumor proportion score (TPS) distribution among PD-L1-tested tumors (*n* = 237)

PD-L1 TPS category	*n*	%
< 1%	63	26.6
1–49%	108	45.6
>=50%	66	27.8

### Associations with PD-L1 high expression

Within the 223 PD-L1-tested tumors with a molecularly evaluable mutually exclusive driver category, PD-L1 high expression was more common in the primary KRAS category than in all other evaluable categories (24/54 [44.4%] vs. 35/169 [20.7%]; OR, 3.06; 95% CI, 1.59–5.89; *p* = 0.001; descriptive prevalence ratio, 2.15). In contrast, TPS > = 50% was infrequent in the primary EGFR category, but this result was based on only two PD-L1-high events (2/26 [7.7%] vs. 57/197 [28.9%]; OR, 0.20; 95% CI, 0.05–0.89; *p* = 0.019; descriptive prevalence ratio, 0.27) (Table 5).

**Table 5:** Associations with PD-L1 high expression (TPS > = 50%)

Analysis/comparison	Study group	Comparator	Effect estimate	*p*-value
Prespecified: KRAS vs. other evaluable primary driver categories	24/54 (44.4%)	35/169 (20.7%)	OR 3.06 (95% CI, 1.59–5.89)	0.001
Prespecified: EGFR vs. other evaluable primary driver categories	2/26 (7.7%)	57/197 (28.9%)	OR 0.20 (95% CI, 0.05–0.89)	0.019
Exploratory adenocarcinoma subset: KRAS vs. other	18/44 (40.9%)	17/94 (18.1%)	OR 3.14 (95% CI, 1.41–6.97)	0.008
Exploratory adenocarcinoma subset: EGFR vs. other	1/23 (4.3%)	34/115 (29.6%)	OR 0.11 (95% CI, 0.01–0.84)	0.009

Analyses were restricted to PD-L1-tested tumors with a molecularly evaluable, mutually exclusive primary driver category (*n* = 223); nine indeterminate and five concomitant/NRAS PD-L1-tested cases were excluded. ORs are unadjusted association measures. Adenocarcinoma analyses are exploratory; EGFR estimates are sparse-event limited. No multiplicity adjustment was applied. Updated Firth sensitivity estimates are in Supporting Table S5

The univariable Firth sensitivity estimates were directionally consistent with the primary 2 × 2 results (KRAS: penalized OR, 3.04; 95% profile CI, 1.59–5.83; *p* < 0.001; EGFR: penalized OR, 0.25; 95% profile CI, 0.05–0.80; *p* = 0.017) (Supporting Table S5). These sensitivity estimates do not address confounding, and the EGFR estimate remains limited by two events.

Within the molecularly evaluable, mutually exclusive PD-L1-tested adenocarcinoma subset (*n* = 138), the same directional pattern was observed. KRAS-mutant tumors were more likely to show TPS > = 50% (18/44 [40.9%] vs. 17/94 [18.1%]; OR, 3.14; 95% CI, 1.41–6.97; *p* = 0.008), whereas EGFR-mutant tumors rarely showed PD-L1 high expression (1/23 [4.3%] vs. 34/115 [29.6%]; OR, 0.11; 95% CI, 0.01–0.84; *p* = 0.009). These analyses were exploratory and event-count limited, particularly for EGFR.

### Smoking history

Smoking history was documented in 69 patients (23.1%) in the retained patient-level export: 45 current, 17 former, and seven never smokers. Documentation was missing or unknown in 230 patients (76.9%). These values replace aggregate counts that could not be reconciled to the retained patient-level source. Smoking was not modeled, and residual confounding by smoking remains possible.

### Representative histology and immunohistochemistry

Representative H&E morphology and immunohistochemical staining patterns for TTF-1, Napsin-A, and PD-L1 (SP263) are shown across increasing PD-L1 TPS categories (approximately 5%, 15%, and > = 50%) (Fig. 1).

## Discussion

This consecutive real-world thoracic oncology cohort provides a practical view of how PD-L1 TPS is distributed across targeted NGS-defined molecular subsets in routine NSCLC diagnosis. The most consistent finding was enrichment of PD-L1 TPS > = 50% among KRAS-mutant tumors. EGFR-mutant tumors showed a lower frequency of PD-L1 high expression, but this observation was limited by sparse events and should be interpreted as directionally supportive rather than definitive. The central message is therefore deliberately cautious: PD-L1 should be interpreted together with driver status, tissue pathway, and testing completeness rather than as an isolated biomarker result.

Our findings are concordant with prior meta-analyses and large real-world studies from other populations, including Asian cohorts and broad international datasets, which have generally reported higher PD-L1 expression in KRAS-mutant NSCLC and lower PD-L1 expression in EGFR-mutant disease [8–12]. The present study therefore does not claim novelty by contradicting those reports. Instead, it contributes a regionally grounded Turkish real-world diagnostic cohort in which SP263-stained slides were re-reviewed, PD-L1 testing availability was analyzed, and DNA/RNA testing limitations were explicitly integrated into interpretation. The applicable population should also be defined carefully: this cohort was male-predominant and KRAS exceeded EGFR, a pattern consistent with a smoking-exposed regional NSCLC population. Driver frequencies and the resulting PD-L1 biomarker distribution may differ substantially in EGFR-predominant populations, including many East Asian cohorts. This practical workflow perspective may help assess whether published biomarker patterns remain recognizable when testing is performed under routine specimen-logistics constraints.

This practical context is important. Current molecular testing recommendations emphasize that NSCLC diagnosis increasingly depends on coordinated morphology, immunohistochemistry, PD-L1 testing, and broad molecular profiling, especially when limited biopsy material must support multiple assays [2–4, 20, 21]. In that setting, PD-L1 immunohistochemistry may be completed before DNA/RNA results are finalized. Our data support a cautious reporting culture in which a high TPS result is not overinterpreted until actionable driver results are reviewed, particularly because targeted treatment implications may outweigh a favorable PD-L1 category in driver-positive disease [18, 19].

The KRAS signal is consistent with prior meta-analyses and large real-world series reporting higher PD-L1 positivity or high expression in KRAS-mutant NSCLC [8–12]. The biological plausibility is also supported by experimental work showing that KRAS pathway activation may contribute to PD-L1 upregulation and immune escape [25, 26]. Importantly, our findings should not be taken to imply that all KRAS-mutant tumors share the same immune phenotype. KRAS-mutant NSCLC is heterogeneous at the allele level, and immune phenotype may be modified by variant subtype and co-alterations such as STK11/LKB1, KEAP1/NFE2L2, and TP53 [13–17, 27–29]. In the present cohort, KRAS G12C and G12V were the most frequent variants, but the study was not powered to compare PD-L1 high expression across individual KRAS variants or co-mutational strata. The patient-level testing-flag audit verified panel counts, but the analytic dataset did not retain comprehensive gene-level co-mutation calls for all KRAS-mutant tumors. Moreover, STK11 and TP53 were included in the 30-gene DNA panel only and therefore could be assessed only in tumors tested on that panel, whereas KEAP1 was absent from both documented DNA panel target lists. KRAS variant data are therefore presented descriptively and should be considered hypothesis-generating.

The EGFR-related pattern is useful for thoracic oncology interpretation but must be framed cautiously. EGFR-mutant tumors were rarely PD-L1 high in the primary and adenocarcinoma-restricted analyses, aligning with previous reports of lower PD-L1 expression and reduced response rates to PD-1 pathway blockade in EGFR-driven NSCLC [8, 9, 18, 19]. Nevertheless, only two EGFR-mutant tumors were PD-L1 high. This finding should therefore be presented as a sparse-data-limited directional observation that reinforces integrated biomarker review rather than stand-alone evidence for clinical decision-making.

A distinctive contribution of the study is the analysis of PD-L1 testing availability. PD-L1 testing was less frequent in externally submitted paraffin blocks than in in-house biopsy or resection/excision specimens, suggesting that missing PD-L1 data may arise from specimen logistics, external block handling, or tissue pathway factors. This issue is often underreported in biomarker studies, yet it directly affects diagnostic completeness and the interpretation of real-world cohorts.

The incomplete smoking history deserves emphasis because smoking is linked to KRAS mutation frequency, tumor mutational burden, and immune phenotype. Only 69 of 299 patients had documented smoking status in the retained patient-level export. Smoking-related results were therefore descriptive, and the primary driver contrasts were intentionally not interpreted as independent biological effects. Residual confounding by smoking remains substantial.

The translational relevance of integrated PD-L1 and driver testing also extends beyond conventional PD-1/PD-L1 blockade. Oncogene-driven NSCLC, particularly EGFR-mutant disease, has shown limited benefit from PD-1 pathway blockade alone despite occasional PD-L1 expression. Emerging strategies are therefore exploring immune mechanisms that are not restricted to the PD-1/PD-L1 axis. One example is CD24-targeted immunotherapy, including ATG-031, a humanized anti-CD24 monoclonal antibody designed to interfere with the CD24/Siglec-10 innate immune checkpoint and enhance macrophage-mediated tumor-cell phagocytosis [30–32]. Preclinical and translational evidence suggests that CD24 targeting may be relevant in EGFR-mutated lung cancer and could potentially complement third-generation EGFR-TKIs.30 These approaches remain investigational and should not be interpreted as clinical recommendations, but they illustrate why PD-L1 expression alone is insufficient to define the immunotherapeutic landscape of driver-positive NSCLC.

Several design features support the main KRAS signal as a biomarker-distribution finding. The cohort was consecutive, molecular testing reflected routine practice, PD-L1 was reported in clinically familiar TPS categories, and archived SP263-stained slides underwent standardized two-pathologist consensus re-review using prespecified criteria. The final driver contrasts were restricted to molecularly evaluable, mutually exclusive categories and were independently reconstructed from the patient-level export. Because independent pre-consensus categories were not retained, the slide re-review should not be read as a quantitative reproducibility analysis.

This study has several limitations that define the boundaries of interpretation. It was retrospective and single-center, and 62 of 299 cases lacked PD-L1 results. The cohort was male-predominant and KRAS exceeded EGFR, so generalizability may differ in EGFR-predominant populations. PD-L1 availability was lower in externally submitted paraffin blocks, suggesting specimen-logistics selection. DNA panel breadth was not uniform, and RNA fusion testing was selective. Eleven patients had indeterminate DNA-driver status after the assay-provenance audit and were excluded from driver analyses; 10 had a negative RNA fusion result without documented DNA testing, and one had an unsupported KRAS label without a retained KRAS-capable assay source. STK11 and TP53 were available only on the 30-gene panel, KEAP1 was absent from both documented DNA panels, and RET coverage differed by assay. These boundaries restrict interpretation of driver-negative, fusion-negative, and co-mutation status.

Other limitations are also important. Molecular data were abstracted from issued clinical reports rather than raw sequencing re-analysis, and the 5% reportable VAF threshold may under-capture low-level subclonal alterations. Smoking history was missing/unknown in 230 of 299 patients in the retained export, preventing reliable adjustment for a key confounder. Stage, treatment sequence, immune checkpoint inhibitor exposure, and survival outcomes were not uniformly available; the study cannot support predictive or prognostic conclusions. The final revision removed previously reported exploratory adjusted models because the corrected molecular set could not be linked to a retained patient-level specimen-type source for independent refitting. The remaining primary and Firth estimates are reproducible but unadjusted. ORs should not be interpreted as relative risks, the binary TPS endpoint discards ordinal information, and formal PD-L1 reproducibility statistics could not be calculated because independent pre-consensus categories were not retained. KRAS variant-level analyses remain descriptive and underpowered.

Future work should move from single-center biomarker distribution toward harmonized clinicogenomic validation. Multi-institutional studies with uniform DNA/RNA panel design, complete gene-level co-mutation and fusion-testing logs, complete smoking and stage annotation, retained independent PD-L1 scores, documented reasons for testing failure, and linked outcome data are needed to determine whether the KRAS and EGFR patterns observed here remain stable across laboratories, platforms, populations, and specimen pathways. Regionally grounded datasets, including recent clinicogenomic evidence from Türkiye, may be especially useful for assessing generalizability in routine practice [22].

## Conclusion

In this consecutive real-world NSCLC diagnostic cohort, PD-L1 TPS > = 50% was enriched among molecularly evaluable primary KRAS-mutant tumors. EGFR-mutant tumors showed a lower PD-L1-high frequency, but this observation was limited by only two events. The findings support integrated interpretation of PD-L1 immunohistochemistry and targeted molecular testing in routine thoracic oncology practice. Because the estimates are unadjusted and outcome data were unavailable, they should be interpreted as biomarker-distribution evidence rather than evidence of immunotherapy benefit or prognosis.

## Supplementary Information

Additional file 1. Supporting Information. Text S1 and Tables S1-S5 provide the case-selection audit, assay content and RNA-only classification, analysis-set definitions, interpretive safeguards, PD-L1 availability comparison, and Firth sensitivity analyses.

## Data Availability

De-identified data supporting the findings of this study are available from the corresponding author on reasonable request, subject to patient confidentiality and institutional and ethics restrictions. Original uncropped microscopy image files underlying Fig. 1 are available from the corresponding author on reasonable request, subject to institutional and ethics restrictions.

## Abbreviations

CI: Confidence interval
FFPE: Formalin-fixed paraffin-embedded
H&E: Hematoxylin and eosin
IHC: Immunohistochemistry
NGS: Next-generation sequencing
NSCLC: Non-small cell lung carcinoma
NOS: Not otherwise specified
OR: Odds ratio
PD-L1: Programmed death-ligand 1
TPS: Tumor proportion score
