# Supplementary material for: PD-L1 expression across molecular driver subsets in non-small cell lung carcinoma: a 299-case real-world thoracic oncology cohort
Source: BMC Cancer. 2026 Jul 30;26:1331. doi: 10.1186/s12885-026-16599-7 (PMC13721590; doi:10.1186/s12885-026-16599-7)
Supplement: Supplementary file 1 [file 12885_2026_16599_MOESM1_ESM.docx]

**Supporting Information**

PD-L1 Expression Across Molecular Driver Subsets in Non-Small Cell Lung Carcinoma: A 299-Case Real-World Thoracic Oncology Cohort

**Text S1. Case-selection and analytic subset definition**

- The analytic export contained 306 specimen-level records. Identity-based de-duplication yielded 299 unique patients; assay flags were consolidated across linked specimens.
- PD-L1 results were available for 237 tumors; 62 lacked an available result. Overall TPS counts remained 63 (<1%), 108 (1-49%), and 66 (>=50%).
- Seven patients with concomitant drivers or isolated NRAS were excluded from mutually exclusive driver analyses.
- Eleven additional patients had indeterminate DNA-driver status: 10 were RNA-fusion negative without documented DNA testing, and one had an unsupported KRAS G12F label without a documented KRAS-capable assay. These 11 were not classified as driver-negative.
- The molecularly evaluable mutually exclusive driver-frequency set therefore contained 281 patients.
- The primary PD-L1 association set contained 223 tumors after excluding nine PD-L1-tested indeterminate cases and five PD-L1-tested concomitant/NRAS cases.
- All adjusted models were removed because the corrected case-level molecular set could not be linked to a retained patient-level specimen-type source for independent refitting.

**Table S1. Targeted DNA/RNA assay content, patient-level counts, and RNA-only classification**

Note: Counts reflect patient-level union of assay flags across repeated specimens. A negative RNA fusion result does not establish absence of DNA sequence variants.

| **Assay component / group** | **Patient-level count** | **Targets or classification** | **Use/notes** |
| --- | --- | --- | --- |
| 9-gene DNA panel | 52/299 (17.4%) | ALK, MET, ROS1, RET, ERBB2, BRAF, EGFR, KRAS, NRAS | All 16 verified KRAS cases in this group had a documented KRAS-capable assay. |
| Comprehensive 30-gene DNA panel | 232/299 (77.6%) | FGFR2, MET, CDK6, SOD2, ESR1, ROS1, RICTOR, TERT, KIT, PDGFRA, PIK3CA, MYD88, ERBB4, IDH1, IDH2, ALK, STK11, ERBB2, TP53, MAP2K1, AKT1, CDK4, GAPDH, PTEN, DDR2, NTRK1, BRAF, EGFR, KRAS, NRAS | All 49 verified KRAS cases in this group had STK11/TP53 panel access; KEAP1 was not covered. |
| Any documented DNA panel | 284/299 (95.0%) | 9-gene or 30-gene DNA panel | The two DNA-panel categories were mutually exclusive after patient-level consolidation. |
| 25-gene RNA fusion panel | 37/299 (12.4%) | NOTCH2, PDGFRA, TERT, PDGFRB, MYB, ETV1, EGFR, MET, BRAF, NRG1, RET, ETV6, NUTM1, ETV4, BRD4, ERG, EWSR1, TFE3, ALK, FGFR2, FGFR3, NTRK1, NTRK2, NTRK3, ROS1; KRAS not included | Selective clinician/pathologist-directed testing; not reflex for all adenocarcinomas. |
| Both DNA and RNA testing | 22/299 (7.4%) | At least one documented DNA panel plus RNA fusion testing | Flags were unioned across linked repeated specimens. |
| RNA-only testing | 15/299 (5.0%) | 4 NTRK2-positive; 10 RNA-negative/no-DNA; 1 unsupported KRAS label/no-DNA source | Four retained as NTRK2-positive; the remaining 11 classified as DNA-driver-status indeterminate, not driver-negative. |

**Table S2. Analysis-set definitions after assay-provenance audit**

Note: Indeterminate cases remained in the overall cohort and PD-L1 distribution but were excluded whenever a defined DNA-driver category was required.

| **Analysis set** | **Included** | **Excluded** | **Final n** |
| --- | --- | --- | --- |
| Overall cohort | All de-duplicated patients | Repeated specimen records | 299 |
| PD-L1 distribution | All patients with a PD-L1 result | 62 without PD-L1 | 237 |
| Mutually exclusive driver frequencies | Molecularly evaluable mutually exclusive categories | 11 indeterminate plus 7 concomitant/NRAS | 281 |
| Primary PD-L1 driver contrasts | PD-L1-tested, molecularly evaluable mutually exclusive categories | 9 tested indeterminate plus 5 tested concomitant/NRAS | 223 |
| Adenocarcinoma-restricted contrasts | Adenocarcinoma subset of primary analysis set | Same molecular exclusions | 138 |

**Table S3. Interpretive safeguards and reproducibility decisions**

| **Issue** | **Final handling** |
| --- | --- |
| RNA-negative/no-DNA cases | Classified as DNA-driver-status indeterminate; never driver-negative. |
| Unsupported KRAS G12F label | Reclassified as indeterminate because KRAS was absent from the RNA panel and no retained KRAS-capable assay source was found. |
| Concomitant/NRAS cases | Excluded from mutually exclusive driver frequencies and primary driver contrasts. |
| Adjusted models | Removed; the corrected molecular set could not be linked to a retained patient-level specimen-type source for independent refitting. |
| Rare-event analysis | Updated univariable Firth estimates retained as reproducible sensitivity analyses. |
| Smoking | Only 69/299 documented in the retained patient export; reported descriptively and not modeled. |
| KRAS heterogeneity | Variant spectrum presented descriptively; no powered allele-specific inferential claim. |
| PD-L1 rescoring | Independent pre-consensus categories were not retained; no kappa/concordance claim. |
| Multiplicity | KRAS/EGFR contrasts prespecified; subgroup analyses exploratory; no multiplicity adjustment. |

**Table S4. Baseline characteristics by PD-L1 testing availability**

Note: The primary-driver p value compares KRAS, EGFR, and other molecularly evaluable mutually exclusive categories only (n = 281). Indeterminate and concomitant/NRAS counts are shown descriptively. Categorical p values are reproducible from the displayed counts; the age comparison uses the displayed summary statistics.

| **Variable** | **PD-L1 tested (n = 237)** | **PD-L1 untested (n = 62)** | **p-value** |
| --- | --- | --- | --- |
| Age, years | 65.7 +/- 8.9 | 65.2 +/- 9.7 | 0.694 |
| Male sex | 198 (83.5%) | 46 (74.2%) | 0.132 |
| Female sex | 39 (16.5%) | 16 (25.8%) |  |
| Adenocarcinoma | 146 (61.6%) | 37 (59.7%) | 0.896 |
| Non-adenocarcinoma | 91 (38.4%) | 25 (40.3%) |  |
| Biopsy specimen | 140 (59.1%) | 29 (46.8%) | 0.001 |
| Resection/excision specimen | 59 (24.9%) | 10 (16.1%) |  |
| Externally submitted paraffin block | 38 (16.0%) | 23 (37.1%) |  |
| Primary KRAS category | 54/223 (24.2%) | 11/58 (19.0%) | 0.180 |
| Primary EGFR category | 26/223 (11.7%) | 12/58 (20.7%) |  |
| Other evaluable mutually exclusive category | 143/223 (64.1%) | 35/58 (60.3%) |  |
| Indeterminate status (descriptive; excluded above) | 9/237 (3.8%) | 2/62 (3.2%) |  |
| Concomitant/NRAS (descriptive; excluded above) | 5/237 (2.1%) | 2/62 (3.2%) |  |

**Table S5. Firth sensitivity analysis for prespecified binary driver contrasts**

Note: Univariable Firth estimates use the same 223-case mutually exclusive molecularly evaluable set as the primary contrasts. Profile-likelihood CIs and penalized likelihood-ratio p values are shown.

| **Contrast** | **Study group** | **Comparator** | **Firth penalized estimate** | **Interpretation** |
| --- | --- | --- | --- | --- |
| KRAS vs. other evaluable primary driver categories | 24/54 high (44.4%) | 35/169 high (20.7%) | Penalized OR 3.04 (95% profile CI, 1.59-5.83); p < 0.001 | Directionally consistent with the prespecified 2 x 2 result. |
| EGFR vs. other evaluable primary driver categories | 2/26 high (7.7%) | 57/197 high (28.9%) | Penalized OR 0.25 (95% profile CI, 0.05-0.80); p = 0.017 | Still based on only two EGFR events; unadjusted and non-causal. |
